# Effectiveness of Dexamethasone in Reducing Arrhythmia in Patients Undergoing Coronary Artery Bypass Grafting

**DOI:** 10.7759/cureus.71746

**Published:** 2024-10-17

**Authors:** Nata Kharimantara Nakamura, Dudy Arman Hanafy, Theresia Feline Husen, Ananda Pipphali Vidya, Albert Tony Lopolisa, Sugisman

**Affiliations:** 1 Adult Cardiac Surgery, National Cardiovascular Center Harapan Kita, Jakarta, IDN; 2 Adult Cardiac Surgery, Faculty of Medicine, University of Indonesia, Jakarta, IDN

**Keywords:** anti-inflammatory, arrhythmia, coronary artery bypass grafting (cabg), dexamethasone, inflammatory marker, off-pump coronary artery bypass (opcab) on-pump coronary artery bypass (oncab)

## Abstract

Introduction

Coronary artery bypass grafting (CABG) carries the risk of postoperative arrhythmias. Our study focusses on the efficacy of dexamethasone in both on-pump CABG (ONCAB) and off-pump CABG (OPCAB).

Methods

This single center randomized control trial was conducted from July 1st, 2018 to January 20th, 2019 in patients undergoing conventional ONCAB and OPCAB at the National Cardiovascular Center Harapan Kita (NCCHK). All arrhythmia incidents were recorded postoperatively with routine monitoring done every hour until the patient was discharged.

Results

One hundred and twenty patients were included in the study and arrhythmias occurred in 24.2% of patients. In the ONCAB groups, there was an association between dexamethasone versus placebo in reducing the incidence of arrhythmias (p = 0.02; OR 0.23 [0.064-0.831]). However, in patients who underwent OPCAB, there was no association between dexamethasone administration and the incidence of arrhythmias (p = 0.347; OR 0.55 [0.157-1.931]). Patients on dexamethasone in both ONCAB and OPCAB groups showed a significant decrease in IL-6, CRP, and procalcitonin (p = 0.001 for all). Overall, arrhythmic subjects had significantly higher levels of inflammatory markers IL-6 (p = 0.013), CRP (p = 0.025), and procalcitonin (p = 0.001).

Conclusion

Dexamethasone reduced postoperative arrhythmias, likely by modulating systemic inflammation, as shown by the decrease in inflammatory markers in ONCAB patients compared to those given a placebo.

## Introduction

Coronary artery bypass grafting (CABG) is a surgical procedure used to bypass an atheromatous blockage in the coronary artery by employing a venous or arterial conduit. The procedure can be performed using various techniques, primarily categorized into conventional, minimally invasive, and robotic methods. The conventional technique is further subdivided into two approaches based on the use of cardiopulmonary bypass: on-pump (ONCAB) and off-pump (OPCAB) [1]. In our center we applied both these conventional sub-techniques. 

CABG carries certain risks that can be mitigated to aid patient recovery and reduce hospital costs. Arrhythmia is one of the most common side effects of cardiac surgery, with data showing that 20% to 40% of patients develop it post-CABG [2-4]. Postoperative arrhythmias following CABG are detrimental to patients, increasing the risk of morbidity and mortality. The occurrence of arrhythmia is associated with a complex inflammatory process triggered by factors such as surgical trauma, blood loss, hypothermia, and ischemia [5,6]. In addition, the cardiopulmonary bypass (CPB) circuit used in ONCAB can further elevate the inflammatory response. During CPB, the heart is often arrested, leading to periods of ischemia and electrolyte imbalances (such as those involving potassium, calcium, and magnesium) that have been shown to alter the heart's rhythm. Moreover, the CPB circuit can cause hypothermia and irritation, which may lead to fluctuations in heart rate and rhythm. On the other hand, although OPCAB results in less systemic inflammation compared to ONCAB, it can still lead to arrhythmias. These arrhythmias may arise due to transient ischemia during graft placement and mechanical stress or manipulation of the heart. However, the incidence and severity of arrhythmias in OPCAB are generally lower than ONCAB [4]. A meta-analysis by Jiang et al. showed that arrhythmias were significantly higher in ONCAB than in OPCAB with a prevalence of 19.1% and 13.1%, respectively [7].

Proper management can reduce postoperative inflammatory processes to mitigate arrhythmias and reduce the risk of death. Corticosteroid use is one of the therapeutic options to prevent postoperative arrhythmia, yet its usage has remained controversial and inconclusive for over three decades. Some studies have investigated the effects of corticosteroids and their potential benefits in reducing arrhythmia incidents, such as the Dexamethasone for Cardiac Surgery (DECS) [8] and Steroids In Cardiac Surgery Trials (SIRS) [9]. However, studies on the use of dexamethasone are limited, particularly in Asia, and previous trials have included all types of cardiac surgery. In addition, another trial performed in our center only focused on major adverse cardiac events and only in OPCAB patients [10]. Hence, this paper aims to clarify the benefits of dexamethasone in reducing arrhythmia during hospitalization for OPCAB and ONCAB, specifically for Indonesian patients. We decided to monitor the incidence of arrhythmias during hospitalization since previous studies have reported that they increase the risk of mortality and other complications [11,12].

## Materials and methods

Study design

This randomized control trial (RCT) was approved by the local ethics committee of the National Cardiovascular Center Harapan Kita, Indonesia, under registration number LBB.01.01/VII/239/KEP.050/2018. Consecutive patients undergoing CABG surgery were enrolled from July 2018 to January 2019.

Inclusion criteria for the study are 1) patients with an indication for CABG surgery, 2) age 18 years or older, and 3) those undergoing elective surgery in the adult cardiac surgery division of the National Cardiovascular Center Harapan Kita (NCCHK). The exclusion criteria are 1) refusal to participate in the study, 2) patients undergoing emergency CABG surgery, 3) patients with signs of systemic inflammation before surgery (fever over 38°C, white cell count ≥15,000/μL), 4) history of cardiopulmonary resuscitation, 5) other concomitant cardiac surgery procedures such as valve and septal repair, 6) New York Heart Association (NYHA) class IV congestive heart failure, kidney failure, respiratory failure, or stroke, 7) re-operation procedures, and 8) a history of steroid consumption before surgery. The power analysis was performed using a power of >80% and a type I error of 5%, with proportions of 33% in the placebo group and 70% in the treatment group, resulting in a minimum required sample size of 25 subjects per group for this study. 

**Figure 1:**
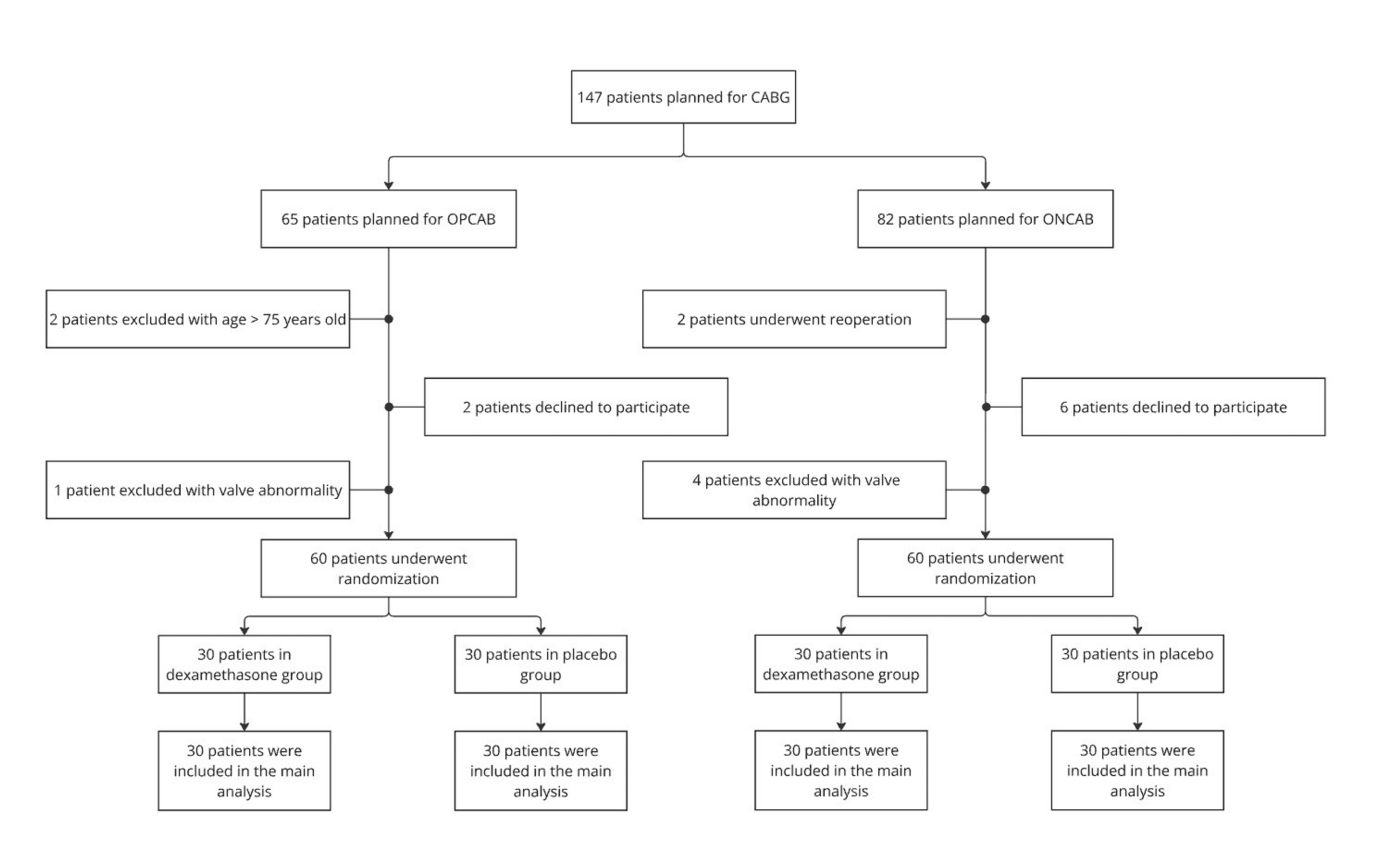
Study design CABG: Coronary artery bypass grafting; ONCAB: Conventional on-pump coronary artery bypass grafting using cardiopulmonary bypass; OPCAB: Off-pump coronary artery bypass grafting.

After applying the inclusion and exclusion criteria, 120 patients passed the criteria and were randomly divided into four groups (ONCAB with dexamethasone, ONCAB with placebo, OPCAB with dexamethasone, and OPCAB with placebo). Informed consent was requested from 65 patients, of which two declined participation, two exceeded the age limit of 75 years, and one was excluded due to valve abnormalities (see Figure 1). The remaining patients were randomly assigned to one of two groups: a dexamethasone group or a placebo group, with 30 patients in each. Randomization was conducted using Research Randomizer (version 4.0) [13]. Both researchers and patients were blinded to the type of intervention. Clinical data were documented using a case report form. 

Patients in the dexamethasone group received a dose of 1 mg per kg body weight (maximum 100 mg) intravenously and patients in the placebo group received normal saline (NaCl 0.9%). ONCAB and OPCAB surgery was performed according to the standard procedure. All the other details that were reported postoperatively have been reported by another study that is also derived from this large RCT [10].

The study's primary endpoint was the incidence of arrhythmia following CABG in all four groups. Arrhythmias occurring at the atrial or ventricular level were recorded postoperatively. Upon transfer from the intensive care unit (ICU) to the general ward, patients were monitored using a 12-lead electrocardiogram (ECG) every hour until they were discharged. Arrhythmias such as atrial fibrillation (AF) lasting for 5 minutes or longer were reviewed by an independent physician, who determined the need for further treatment. In contrast, ventricular tachycardia (VT) or ventricular fibrillation (VF) with or without AF was automatically recorded and treated.

For the secondary outcome, which is the comparison between inflammatory markers (IL-6, CRP, and procalcitonin) and arrhythmia incidence, these markers were reported postoperatively with a minimum of 18 hours. All the patients are followed up during the hospitalization period.

Statistical analysis

The distribution of the data was assessed for normality using the Kolmogorov-Smirnov test. Normally distributed continuous variables are presented as mean ± standard deviation (SD), whereas non-normally distributed data are presented as median (range). Group differences for normally distributed continuous variables were evaluated using the independent T-test; for non-normally distributed variables, the Mann-Whitney U test was used. Categorical variables are presented as frequencies and percentages and were compared using the Chi-square test or Fisher's exact test (in case of expected count <5 or more than 20%). For numerical data across multiple groups, ANOVA was used for normally distributed data, and the Kruskal-Wallis test was used for non-normally distributed data. Propensity score matching was not applied to parameters with significant differences, as it did not alter the significance. Survival analysis was conducted using Kaplan-Meier curves, with Log-Rank test comparisons. A p-value of <0.05 was considered statistically significant. All statistical analyses were performed using IBM SPSS Statistics for Windows, Version 26 (Released 2019; IBM Corp., Armonk, New York, United States).

## Results

A total of 120 patients were enrolled and the four groups had 30 participants each. The groups were dexamethasone (ONCAB and OPCAB) versus placebo (ONCAB and OPCAB). There were no patients lost to follow-up and all patients were analyzed. All groups were comparable with no statistical differences in baseline demographics (age, preoperative ejection fraction, body mass index, gender, comorbidities, arrhythmia type, and operation time). Baseline characteristics can be seen in Table 1. 

**Table 1:** Baseline characteristics of patients from all groups ONCAB: Conventional on-pump coronary artery bypass grafting using cardiopulmonary bypass; OPCAB: Off-pump coronary artery bypass grafting; IQR: Interquartile range; CK-MB: Creatinine kinase-MB; IQR: Interquartile range; Parametric values are presented with mean ± standard deviation (SD); Non-parametric values are presented with a median (Interquartile range). *Analyzed using ANOVA;**Analyzed using the Kruskal-Wallis test;***Analyzed using the Chi-square test.

	ONCAB Dexamethasone (n=30)	ONCAB Placebo (n=30)	OPCAB Dexamethasone (n=30)	OPCAB Placebo (n=30)	p-value
Age, mean ± SD	58.37 ± 6.84	58.87 ± 7.77	61.77 ± 8.11	57.6 ± 7.92	0.173*
Preoperative ejection fraction, median (IQR)	62 (49.5 - 66.75)	60 (52 - 70.5)	58.5 (44.75 - 68.5)	58.5 (51.75 - 64)	0.534**
Body mass index, mean ± SD	26.9 ± 3.14	26.66 ± 4.23	25.21 ± 3.76	25.94 ± 3.89	0.301*
Male, n (%)	25 (83.3)	27 (90)	22 (73.3)	25 (83.3)	0.457***
Female, n (%)	5 (16.7)	3 (10)	8 (26.7)	5 (16.7)	
Hypertension, n (%)	27 (61.4)	28 (58.3)	24 (57.1)	27 (64.3)	
Type II diabetes, n (%)	17 (38.6)	20 (41.7)	18 (42.9)	15 (35.7)	0.619***
Operation time (minutes), mean ± SD	215.9 ± 26.4	214.9 ± 29.24	199.9 ± 34.9	214.93 ± 43.44	0.212*
CK-MB pre-operation, median (IQR)	14.5 (11 - 57)	15.5 (10 - 24)	19 (11 - 45)	17 (10 - 29)	0.17**

Primary outcome

Arrhythmia incidence in ONCAB placebo group was higher compared to ONCAB dexamethasone (40.7% vs 13.3%, respectively; p = 0.02; OR 4.33 [1.20-15.61]) (Table 2).

**Table 2:** Postoperative arrhythmia incidence in the ONCAB groups ONCAB: Conventional on-pump coronary artery bypass grafting using cardiopulmonary bypass; CI: Confidence interval; OR: Odds ratio

Intervention	Arrhythmia		p-value	OR (95% CI)
	Yes	No		
ONCAB Placebo, n (%)	12 (40.7)	18 (60.0)	0.02*	4.33 (1.20 - 15.61)
ONCAB Dexamethasone, n (%)	4 (13.3)	26 (86.7)		

However, arrhythmia incidence was comparable in the OPCAB placebo and OPCAB dexamethasone groups (26.7% vs 16.7%, respectively; p = 0.347; OR 1.82 [0.52-6.38]) (Table 3).

**Table 3:** Postoperative arrhythmia incidence in the OPCAB groups OPCAB: Off-Pump coronary artery bypass grafting; CI: Confidence interval; OR: Odds ratio; *Analyzed with the Chi-square test.

Intervention	Arrhythmia		p-value	OR (95% CI)
	Yes	No		
OPCAB Placebo, n (%)	8 (26.7)	22 (73.3)	0.347*	1.82 (0.52-6.38)
OPCAB Dexamethasone, n (%)	5 (16.7)	25 (83.3)		

In addition, arrhythmia types did not differ between the two intervention groups, for both ONCAB and OPCAB techniques (Table 4 and Table 5).

**Table 4:** Types of postoperative arrhythmias in the ONCAB groups ONCAB: Conventional on-pump coronary artery bypass grafting using cardiopulmonary bypass; AF: Atrial fibrillation; VT/VT: Ventricular tachycardia/ventricular fibrillation. *Analyzed using the Chi-square test; **Analyzed using the Fisher's exact test.

Arrhythmia	ONCAB Placebo, n (%)	ONCAB Dexamethasone, n (%)	p-value
AF	7 (23.3)	3 (10)	0.166*
VT/VF	4 (13.3)	0 (0)	0.112**
VT/VF with AF	1 (3.3)	1 (3.3)	1.0*

**Table 5:** Types of postoperative arrhythmias in the OPCAB groups OPCAB: Off-pump coronary artery bypass grafting; AF: Atrial fibrillation; VT/VT: Ventricular tachycardia/ventricular fibrillation. *Analyzed using the Chi-square test.

Arrhythmia	OPCAB Placebo, n (%)	OPCAB Dexamethasone, n (%)	p-value
AF	5 (16.7)	4 (13.3)	0.718*
VT/VF	2 (6.7)	1 (3.3)	0.554*
VT/VF with AF	1 (3.3)	0 (0.0)	0.313*

Secondary outcome

Comparison between inflammatory markers (IL-6, CRP, and procalcitonin) and arrhythmia incidence showed that inflammatory markers were significantly higher in patients with arrhythmia (IL-6: p=0.013, CRP: p=0.025, procalcitonin: p=0.001) (Table 6).

**Table 6:** Inflammatory markers in patients with or without postoperative arrhythmia All data are presented with the median (interquartile range). IL-6: Interleukin 6, CRP: C-reactive protein. **Analyzed using the Mann-Whitney U test.

Postoperative inflammatory markers	Arrhythmia		p-value
	Yes	No	
IL-6 (pg/mL)	382 (165.5 - 557)	184 (110 - 361)	0.013**
CRP (mg/L)	98 (58.5 - 158)	71 (47 - 124)	0.025**
Procalcitonin (ng/mL)	5.06 (2.28 - 8.23)	2.19 (1.08 - 4.05)	0.001**

Compared to CRP, IL-6 and procalcitonin levels were more elevated. In addition, the ONCAB placebo group had the highest levels of IL-6, CRP, and procalcitonin compared to all other groups. There was no significant difference in the inflammatory marker levels between the ONCAB placebo and OPCAB placebo groups. However, inflammatory marker levels were significantly lower in both ONCAB dexamethasone and OPCAB dexamethasone groups compared to the ONCAB placebo group (p<0.001) (Tables 7-9).

**Table 7:** Postoperative CRP levels across all four groups ONCAB: Conventional on-pump coronary artery bypass grafting using cardiopulmonary bypass; OPCAB: Off-pump coronary artery bypass grafting; CRP: C-reactive protein; *Analyzed using the Kruskal-Wallis test; **Analyzed using the Mann-Whitney U test.

Group	CRP (mg/L)	p-value	p-value between groups
ONCAB Placebo	127.5 (68.75 - 189.5)	<0.001*	Control
ONCAB Dexamethasone	54.5 (33 - 88.75)		<0.001**
OPCAB Placebo	122.5 (78.75 - 151.25)		0.636**
OPCAB Dexamethasone	64.5 (43,.5 - 85.5)		<0.001**

**Table 8:** Postoperative procalcitonin levels across all four groups ONCAB: Conventional on-pump coronary artery bypass grafting using cardiopulmonary bypass; OPCAB: Off-pump coronary artery bypass grafting; *Analyzed using the Kruskal-Wallis test; **Analyzed using the Mann-Whitney U test.

Group	Procalcitonin (ng/mL)	p-value	p-value between groups
ONCAB Placebo	5.14 (3.42 - 8.42)	<0.001*	Control
ONCAB Dexamethasone	1.09 (0.77 - 1.74)		<0.001**
OPCAB Placebo	4.5 (2.3 - 8.3)		0.530**
OPCAB Dexamethasone	1.77 (0.94 - 3.05)		<0.001**

**Table 9:** Postoperative IL-6 levels across all four groups ONCAB: Conventional on-pump coronary artery bypass grafting using cardiopulmonary bypass; OPCAB: Off-pump coronary artery bypass grafting; IL-6: Interleukin 6; *Analyzed using the Kruskal-Wallis test; **Analyzed using the Mann-Whitney U test.

Group	IL-6 (pg/mL)	p-value	p-value between groups
ONCAB Placebo	440.5 (317.75 - 687.5)	<0.001*	Control
ONCAB Dexamethasone	114 (84.5 - 157)		<0.001**
OPCAB Placebo	363.5 (230 - 560.5)		0.165**
OPCAB Dexamethasone	128 (92.5 - 231.5)		<0.001*

In addition to significantly lower levels of inflammatory markers, the overall hospital stay for patients in the dexamethasone groups was significantly shorter than the placebo groups. However, the ICU stay was similar in all groups (Table 10).

**Table 10:** Hours in the ICU and overall hospital stay across all four groups ONCAB: Conventional on-pump coronary artery bypass grafting using cardiopulmonary bypass; OPCAB: Off-pump coronary artery bypass grafting; ICU: Intensive care unit; Non-parametric values are presented with a median (IQR or interquartile range); **Analyzed using the Kruskal-Wallis test.

	ONCAB Dexamethasone	ONCAB Placebo	OPCAB Dexamethasone	OPCAB Placebo	p-value
ICU stay (hours), median (IQR)	17 (11 - 30)	19 (12 - 155)	18 (12 - 32)	19 (13 - 168)	0.05**
Hospital stay (days), median (IQR)	5 (5 - 8)	7 (6 - 30)	5 (5 - 8)	6.5 (5 - 30)	<0.05**

## Discussion

In this study, we evaluated the effect of dexamethasone on preventing the incidence of arrhythmia in patients undergoing CABG. Subjects in the dexamethasone group experienced fewer arrhythmias, consistent with the findings of a meta-analysis by Ho et al. [14], who reported that corticosteroids tend to reduce the incidence of new-onset AF. The overall incidence of arrhythmias was lower, though the significance of this reduction varied between the ONCAB and OPCAB groups. Notably, ONCAB demonstrated a significantly different outcome compared to OPCAB, suggesting that dexamethasone has a more pronounced effect on patients undergoing the former. This finding is likely related to the heightened inflammatory response associated with ONCAB compared to OPCAB. During the use of CPB, blood comes into contact with the non-endothelial surfaces of the bypass circuit, which activates the complement system and other immune pathways, leading to a systemic inflammatory response. Additionally, the mechanical stress and shear forces generated by the pump, along with ischemia-reperfusion injury when normal circulation is restored, further amplify the release of proinflammatory cytokines and mediators. This heightened inflammatory state contributes to tissue injury, oxidative stress, and various postoperative complications, including arrhythmias [7,15].

In addition to inducing a systemic inflammatory response, ONCAB is associated with several other factors that contribute to a higher incidence of postoperative arrhythmias. These include significant electrolyte imbalances, particularly involving potassium and magnesium, which disrupt the myocardial electrical environment and predispose patients to arrhythmias like AF. ONCAB also increases the risk of myocardial ischemia due to aortic clamping and ischemia-reperfusion injury, leading to myocardial cell damage that fosters arrhythmogenesis. Furthermore, the mechanical manipulation of the heart and exposure to the CPB circuit during ONCAB directly irritate the cardiac conduction system. These factors coupled with temperature fluctuations caused by hypothermia and subsequent rewarming can collectively explain the higher rate of postoperative arrhythmias observed with ONCAB compared to OPCAB [16].

Moreover, we analyzed each type of arrhythmia and our study showed that both interventions showed similar results in both techniques in terms of statistics where there was some effect, but with no significant result. The incidence of AF was lower in the dexamethasone groups than in the placebo groups, although it was statistically insignificant. This result aligns with other studies, which have also suggested that the incidence of postoperative AF following CABG is lower in patients treated with corticosteroids [17-19]. In our study, two subjects in the OPCAB placebo group experienced VT/VF, compared to only one subject in the OPCAB dexamethasone group, and four subjects in the ONCAB placebo group experienced VT/VF, compared to zero subjects in the ONCAB dexamethasone group. Although this difference was not statistically significant, it suggests a considerable reduction in arrhythmia incidence (absolute relative risk reduction was 10%). This result aligns with the studies by researchers from Japan, such as Rimmelé et al. [20], who found that administering corticosteroids during OPCAB reduced the incidence of postoperative AF, although they did not provide information on the incidence of VT/VF. There are also limited studies on the ONCAB and incidence of VT/VF. Although our study provides statistically insignificant results for different types of arrhythmias, the result is significant when we combine all types of arrhythmias in the ONCAB group. This leads to the probability that the insignificance of each arrhythmia may be caused by the smaller sample size. Different patient demographics might also influence the results.

Dexamethasone administration also decreases the inflammatory response. This inflammatory response was objectively assessed by examining markers such as IL-6, CRP, and procalcitonin. These markers were selected based on their diagnostic capabilities and cost efficiency. Using the Kruskal-Wallis analysis test and by keeping the ONCAB placebo group as a reference, we found that IL-6, CRP, and procalcitonin levels differed significantly among the four treatment groups (p < 0.001). The placebo group exhibited significantly higher inflammation compared to the dexamethasone groups, which is consistent with the findings of a meta-analysis by Liu et al. [21].

Furthermore, the ONCAB placebo group had the highest levels of IL-6, CRP, and procalcitonin compared to the other groups. There were no significant differences in these markers between the ONCAB placebo group and the OPCAB placebo group, consistent with other studies that have indicated that the inflammatory response is due to surgery itself rather than the use of an extracorporeal circuit and have reported no significant differences in IL-6 levels between ONCAB and OPCAB groups [22,23]. Although, statistically insignificant, the levels of inflammatory markers were still higher in the ONCAB placebo group. This has probably contributed to the more profound effect seen with dexamethasone on arrhythmia incidence, explaining its statistically significant effect in the ONCAB group compared to the OPCAB group. 

The inflammatory results obtained in this study are consistent with other research, which has demonstrated a correlative relationship between the systemic inflammatory marker IL-6 and the incidence of arrhythmias [24-26]. A study titled the Heart and Soul Study also found that IL-6 is closely associated with the incidence of arrhythmias, particularly atrial arrhythmias such as AF [27]. Inflammatory cytokines may cause arrhythmias by altering ion channel function in heart cells, thereby disrupting the cardiac conduction system and leading to arrhythmias at both the atrial and ventricular levels. AF is also closely related to the inflammatory marker CRP [28].

Limitations

The study is limited by its relatively small sample size, making it suitable as a preliminary study for future, larger validation studies, particularly in the Indonesian population. Additionally, its retrospective design, relying on data from a previously conducted RCT, and the short follow-up period, confined to the duration of hospitalization, further constrain its findings. Future research should involve longer follow-up periods, larger sample sizes, and a more diverse population from multiple centers.

## Conclusions

Dexamethasone administration was associated with a significant reduction in the incidence of postoperative arrhythmias in Indonesian patients undergoing CABG. This effect is likely attributable to dexamethasone's ability to modulate the systemic inflammatory response, as evidenced by a marked decrease in postoperative inflammatory markers in patients who received dexamethasone during CABG, both in ONCAB and OPCAB, compared to those who received a placebo. These findings underscore the potential of dexamethasone in not only controlling inflammation but also to mitigating associated complications such as arrhythmias in the postoperative setting.
